# Conifer Needle Epidermal Imprints for Stomatal Size and Density Measurements

**DOI:** 10.1002/cpz1.70502

**Published:** 2026-10-23

**Authors:** Lena Sachsenmaier, Selina Franz, Anja Kahl, Lena Kretz, Christian Wirth

**Affiliations:** ^1^ German Centre for Integrative Biodiversity Research (iDiv) Halle‐Jena‐Leipzig Germany; ^2^ Systematic Botany and Functional Biodiversity Leipzig University Leipzig Germany; ^3^ Max Planck Institute for Biogeochemistry Jena Germany

**Keywords:** conifer, guard cell length, imprint, stomata, stomatal density

## Abstract

Stomata play a central role in regulating plant water loss and gas exchange, making functional trait data on stomatal density and size increasingly relevant in the context of climate change. Expanding comparative datasets for these traits requires simple, low‐cost methods that can be applied consistently across species and environments.

However, while simple stomatal imprint techniques using nail varnish and adhesive tape are widely established for angiosperms, their application to conifer needles is challenging. Stomata in conifers are typically sunken within epistomatal chambers and occluded by densely meshed wax tubules, which can prevent accurate replication of stomatal complexes in epidermal imprints and thereby limit reliable measurement of stomatal size. In addition, stomata are often heterogeneously distributed across needle surfaces and may occur on multiple surfaces depending on species, requiring tailored sampling approaches for accurate density estimation.

Consequently, reliable measurement of stomatal traits in conifers would require chemical processing and advanced microscopy, making these methods time‐intensive, expensive and less suitable for large‐scale comparative studies. Therefore, we lack an approach for conifers that generates high‐quality trait data while remaining fast, simple, and comparable to established angiosperm methods.

Here, we present a straightforward protocol for preparing stomatal imprints in conifer needles based on standard methods for angiosperm species but including simple preprocessing steps to improve imprint consistency and quality. We further provide guidance for identifying, imaging, and quantifying stomata while accounting for their species‐specific spatial distribution on needle surfaces. The protocol enables reliable measurements of stomatal size and density using basic laboratory equipment and standard light microscopy. We demonstrate its applicability across 12 northern hemisphere conifer species, highlighting its suitability for comparative trait‐based studies. © 2026 The Author(s). *Current Protocols* published by Wiley Periodicals LLC.

**Support Protocol 1**: Sampling conifer needles for stomatal traits

**Basic Protocol 1**: Epidermal imprints using nail varnish

**Basic Protocol 2**: Measurement of stomatal density and size

## Introduction

Stomata play a key role in regulating plant water loss during drought by closing under water‐limited conditions to reduce transpiration and reopening after stress to resume photosynthetic carbon assimilation. The morphology and distribution of stomata are species‐specific and influence stomatal function and behaviour. Stomatal density has been discussed as a determinant of maximum stomatal conductance (de Boer et al., 2016; Franks & Farquhar, 2007), while stomatal size has been suggested to influence the speed of stomatal opening and closing (Drake et al., 2013; Elliott‐Kingston et al., 2016; Lawson & Vialet‐Chabrand, 2019). This makes functional trait data on stomatal density and size increasingly important for understanding and predicting plant responses to climate change (Bertolino et al., 2019; Franks et al., 2009). However, most empirical work on stomatal traits has focused on angiosperms, while gymnosperms remain underrepresented in trait datasets. This is a critical gap given that conifers are key components of large forest ecosystems across the boreal, temperate, and montane zones and have distinct stomatal morphology and hydraulic strategies compared to broadleaved species (Bouche et al., 2014; Brodribb et al., 2012). The scarcity of conifer stomatal trait data likely reflects methodological challenges, as stomata are arranged in species‐specific patterns in distinct bands and rows rather than uniformly across the leaf surface (Rudall et al., 2013), and epicuticular wax layers further obscure stomatal boundaries (Brodribb & Hill, 1997), complicating both density and size measurements. These challenges highlight the need for a standardized protocol for measuring stomatal traits in conifers that is easy to apply, cost‐effective, applicable across a broad range of conifer species, and comparable to angiosperm measurements.

Stomata are small pores on plant surfaces bounded by a pair of guard cells, often flanked by subsidiary cells; together, these structures form the stomatal complex. In conifers, stomata are deeply sunken into the needle surface, sitting in longitudinal furrows that form stomatal antechambers (Šantrůček, 2022), creating a thick boundary layer that drastically reduces water loss. Further, in many conifers, the subsidiary cells do not simply sit adjacent to the guard cells but arch over them, leaving only a narrow passage leading to the pore (Gray et al., 2020). For some species, the subsidiary cells even form a distinct, raised, ring‐like structure known as the Florin ring (Florin, 1931), which acts like a tiny collar to further protect the pore. Typically, conifer species have their antestomatal chambers filled partly or completely with porous plugs of wax (Figure 1) (Brodribb & Hill, 1997; Jeffree et al., 1971; Kim et al., 2010, 2011), which likely evolved to prevent the stomatal pore from being blocked by liquid water films (Šantrůček, 2022).

**Figure 1:**
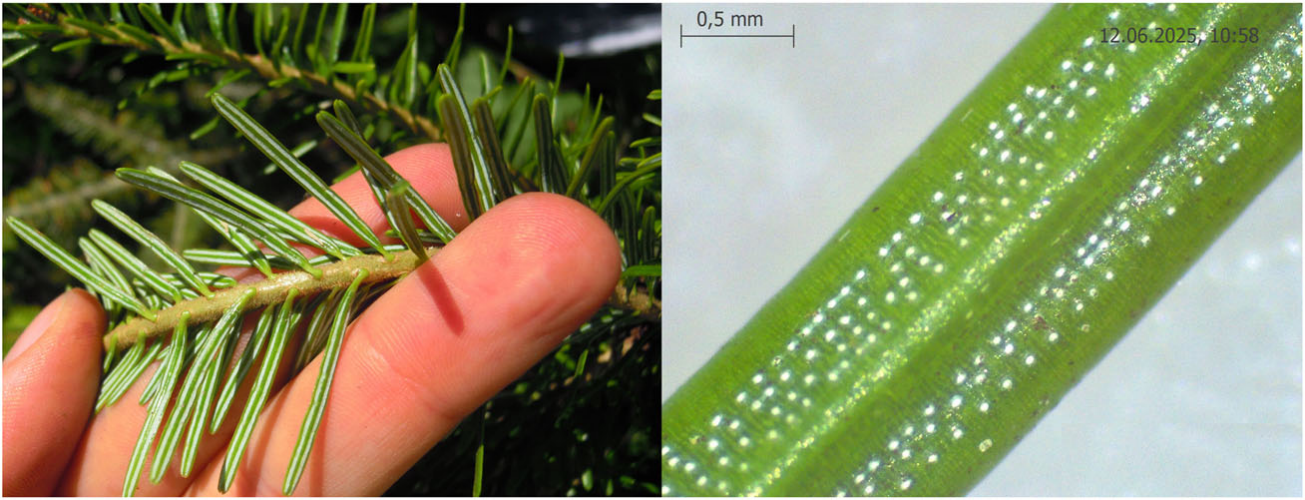
Epicuticular wax structures on conifer needles. Left: The abaxial needle surface of *Abies alba* showing the characteristic white epicuticular wax bands, visible at the macroscopic scale. Right: Image of a *Picea abies* needle surface showing wax plugs filling the sunken stomatal antechambers, photographed under a stereomicroscope equipped with a camera (Di‐Li 2009‐16, Distelkamp‐Electronic Kaiserslautern, Germany).

These specific architectural features necessitate modifications to standard, low‐cost methods for measuring stomatal traits. Traditional leaf‐surface imprint techniques commonly used for angiosperms—such as nail varnish replicas viewed under a standard light microscope—fail for two primary reasons: first, the presence of heavy wax plugs and layers conceals the underlying elements of the stomatal complex from detection; second, because the stomata are deeply sunken, the nail varnish cannot effectively reach them, leaving hollow holes in the imprint where the stomata should be. Furthermore, quantifying stomatal density is complicated by the needle's spatial organization. Because stomata are strictly confined to localized longitudinal bands rather than evenly distributed, researchers must clearly specify whether density is reported relative to the total needle surface area, the surface area that generally harbours stomatal bands (stomata‐bearing surface), or the stomatal bands themselves. This requires prior knowledge of the species‐specific leaf anatomy, whether the needle is hypostomatic (stomata only on the abaxial side), amphistomatic (on both abaxial and adaxial sides), or possesses stomata on more leaf sides because of a triangular, quadrangular, or circular cross‐section, to determine precisely where the stomatal bands are located and where measurements should be taken.

Consequently, the accurate assessment of conifer stomatal morphology has traditionally required labour‐ and cost‐intensive techniques such as chemical tissue clearing, mechanical cross‐sectioning, and high‐resolution scanning electron microscopy (Finsinger & Tinner, 2020; Kim et al., 2010; Kim, Lee, Bae, & Kim, 2011; Lacourse et al., 2016). For ecological studies, which often require high‐throughput trait measurements across numerous leaves per species, such techniques are less practical. Conversely, lower‐cost alternatives remain heavily compromised; many studies take only rough measurements of the visible wax plugs under a stereomicroscope (Figure 1) or simply count the hollow gaps left behind in nail varnish imprints where stomata are assumed to have been.

To resolve this issue, we present simple preprocessing techniques and practical imprinting tips that improve imprint image quality under a standard light microscope. By measuring guard cell length and guard cell pair width alongside the overall length and width of the entire stomatal complex (including the subsidiary cells), researchers can obtain a highly precise overview of stomatal traits, even though our protocol cannot completely overcome the physical barrier of overarching subsidiary cells, which occur in some species. Since a two‐dimensional nail varnish imprint cannot isolate the distinct cellular layers of a three‐dimensional stomatal complex (Finsinger & Tinner, 2020), only maintaining absolute methodological consistency—i.e., utilising a single observer and a standardized focusing depth—ensures that the resulting data remain highly comparable across measurements and improves comparability across species, including angiosperms.

Additionally, we provide a reference guide for 12 distinct conifer species (8 genera) that details needle geometric shapes, identifies which surfaces bear stomata, maps their localized distribution, and provides explicit instructions on where to apply the imprints, offering an accessible, low‐cost framework for assessing conifer stomatal traits.

This article presents a comprehensive step‐by‐step guide to analysing stomatal traits on conifer needles. It covers needle sampling (Support Protocol 1), creating epidermal surface imprints (Basic Protocol 1), and measuring stomatal density and size from those imprints (Basic Protocol 2). To ensure reproducibility, we also provide a reference dataset of 12 conifer species and imagery, allowing users to confidently cross‐check results. Beyond its use as a practical reference, this dataset provides ground‐truth material for developing, validating, and benchmarking automated image‐analysis pipelines and emerging machine‐learning approaches for stomatal trait quantification (Fetter et al., 2019; Tan et al., 2024).

## Support Protocol 1 SAMPLING CONIFER NEEDLES FOR STOMATAL TRAITS

In this protocol, we provide detailed, step‐by‐step instructions for sampling conifer needles for later epidermal imprinting (see Basic Protocol 1) and offer guidance to avoid common pitfalls.

### Materials

Resealable thick plastic sampling bags (e.g., zip‐lock bags), pre‐labelled
Laboratory tissues or moist paper towels for sample hydration
Ladder or canopy access equipment, if required
Pruning shears or telescopic pole pruner for twig collection
Spray bottle containing 500 ml of distilled water
Insulated cool box or portable cooler with ice packs
4°C refrigerator

1 Prepare the sampling bags by placing one sheet of dry lab tissue into each bag.
  The use of thick plastic bags prevents the sharp conifer needles from puncturing the material and breaking the humidity seal.
2 Select a top‐canopy, sun‐exposed branch section with the needle cohort from last year (1‐year‐old cohort) that is fully mature.
  Bud scale scars on the twig serve as anatomical markers to identify the boundaries between growth years. Immature current‐year needles and older interior needles (≥3 years old) are generally avoided due to potential degradation. For deciduous conifers like Larix decidua, sampling should occur late enough in the season for the needles to reach full maturity; at our study site in Central Europe, this is typically from mid‐June onward. Avoid newly developing long shoots, which carry younger needles.
3 Cut a small, intact twig section (rather than plucking single needles) containing enough healthy needles to allow for multiple subsequent imprint attempts.
  Healthy samples are verified to be free from any visible signs of fungal infection, galls, or herbivory.
4 Place the cut twig carefully into the prepared sampling bag and spray the lab tissue with distilled water to create a humid environment.
5 Seal the bag immediately and position it securely inside a field cooler for transport.
  Direct contact between the conifer twigs and the cooling elements must be avoided, because freezing may rupture the needle cells and compromise epidermal integrity.
6 Transfer all sampling bags into a refrigerator maintained at 4°C for a maximum storage time of 1 week.
  Although storage potential varies by species, delaying the imprint process will cause imprint quality to decline and increase the risk of fungal growth appearing in your final imprints.
7 Proceed with the preparation of epidermal imprints using nail varnish according to Basic Protocol 1.

## Basic Protocol 1 EPIDERMAL IMPRINTS USING NAIL VARNISH

In this protocol, we provide detailed, step‐by‐step instructions to optimize the standard nail varnish imprinting method for challenging conifer needles. Specifically, we show how to preprocess needles to remove obscuring epicuticular waxes, create clean epidermal imprints, and securely mount these transparent replicas onto microscope slides for storage and subsequent analysis. This method is deliberately designed to be simple and highly cost‐effective, allowing it to be carried out in basic field laboratories using common, readily available laboratory and consumer materials, without the need for specialized equipment or chemicals. When conducted properly, you can expect to obtain high‐contrast surface replicas under a standard light microscope. These imprints will provide a reliable, reproducible framework for quantifying stomatal density and size across diverse conifer species.

### Materials

Conifer needle samples
250‐ml beaker
Distilled water (250 ml)
Ordinary mild washing‐up liquid (e.g., FIT hand dishwashing detergent, fit GmbH, Germany)

Temperature‐controlled water bath
Tweezers
Cotton swabs (one per sample)
Adhesive tape (e.g., tesafilm hand‐tearable, TESA SE, Germany)
Transparent nail varnish (e.g., Manhattan Last & Shine nail polish, Coty, USA)
Microscope slides (one per sample)
Slide label or marker
Stereomicroscope
Light microscope

#### Species‐specific leaf surface selection

1 Determine the correct species‐specific surface(s) for imprinting prior to any further processing to ensure that the target epidermis is correctly exposed and sampled. Species‐specific instructions are provided in Table 1, their cross‐sectional geometry is shown in Figure 2, and a graphical helper for identifying imprint locations is provided in Figure 3.
  Conifer needles vary in cross‐sectional geometry from flattened to cylindrical forms (Figure 2). Stomata may occur on one or multiple of these surfaces, depending on the species. Therefore, the number and position of required imprints must be adjusted accordingly, and steps 2–13 must be repeated separately for each relevant surface. See Table 1 for species‐specific details on how many imprints are required and how and where they should be taken.

**Table 1:** Species‐specific methodology for stomatal imprints and stomata counting, depending on needle cross‐section and stomata arrangements

Species name	Needle cross‐section (Figure 2)	Stomata arrangement (Figure 10)	Imprint specifications	Location for counting stomata
*Abies alba* Mill.	Flat	Abaxial, in 2 bands	Abaxial, full width	Across full width (including stomata‐free zones) at 3 random spots along length
*Abies grandis* (Douglas ex D.Don) Lindl.	Flat	Abaxial, in 2 bands	Abaxial, full width	Across full width (including stomata‐free zones) at 3 random spots along length
*Cedrus deodara* (Roxb. ex D.Don) G.Don	Roundish	In equally distributed rows	Random location	3 random spots along length
*Larix decidua* Mill.	Flat	In rows with different spacing	Random surface, full width	Across full width (including stomata‐free zones) at 3 random spots along length
*Picea abies* (L.) H.Karst.	Diamond	In rows with different spacing	Random surface, full width	Across full width (including stomata‐free zones) at 3 random spots along length
*Picea sitchensis* (Bong.) Carrière	Diamond	Adaxial in 1–3 rows, abaxial in 2 bands	Adaxial and abaxial, each full width	For each (adaxial & abaxial): across full width (including stomata‐free zones) at 3 random spots along length
*Pinus nigra* J.F.Arnold	Semicircle (flat‐bottom)	In equally distributed rows	Random location	3 random spots along length
*Pinus ponderosa* Douglas ex C.Lawson	Semicircle (triangular bottom)	In equally distributed rows	Random location	3 random spots along length
*Pinus sylvestris* L.	Semicircle (flat‐bottom)	In equally distributed rows	Random location	3 random spots along length
*Pseudotsuga menziesii* (Mirb.) Franco	Flat	Abaxial, in 2 bands	Abaxial, full width	Across full width (including stomata‐free zones) at 3 random spots along length
*Taxus baccata* L.	Flat	Abaxial, equally (except midvein)	Abaxial, full width	3 random spots along length
*Tsuga canadensis* (L.) Carrière	Flat	Abaxial, in 2 bands	Abaxial, full width	Across full width (including stomata‐free zones) at 3 random spots along length

**Figure 2:**
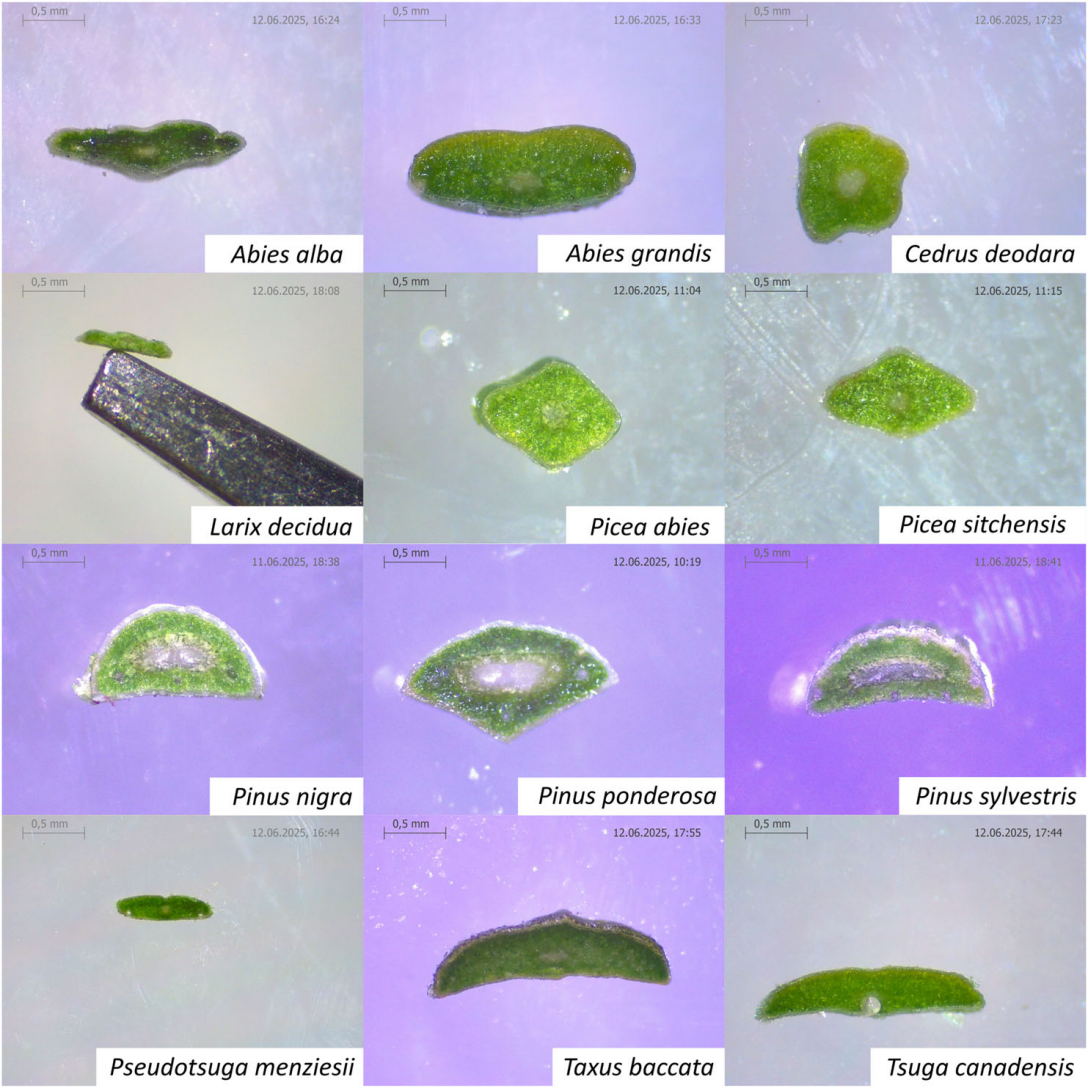
Needle cross‐sections of 12 conifer species, photographed under a stereomicroscope equipped with a camera (Di‐Li 2009‐16, Distelkamp‐Electronic Kaiserslautern, Germany).

**Figure 3:**
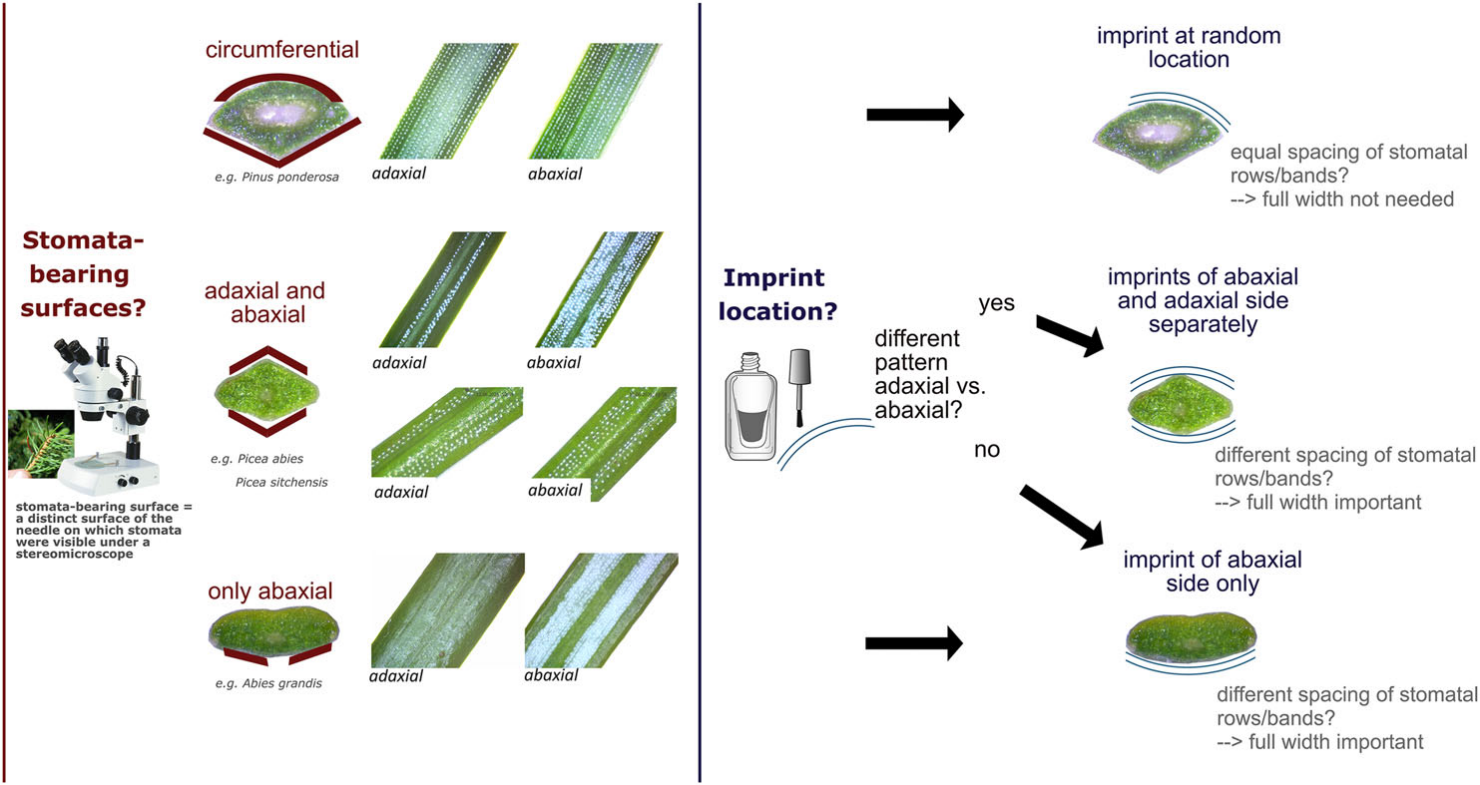
Identification of stomata‐bearing surfaces and selection of nail‐varnish imprint locations. A stomata‐bearing surface is defined as a distinct needle surface on which stomata are visible under a stereomicroscope. Depending on stomatal distribution, needles may have stomata‐bearing surfaces around the circumference, on the abaxial surface only, or on both the adaxial and abaxial surfaces. Where both surfaces bear stomata, their distribution is assessed to determine whether separate imprints are taken from both surfaces or from the abaxial surface only. Needle cross‐sections and stereomicroscope images illustrate the identification of stomata‐bearing surfaces. On the right, the marked stripe indicates the location selected for the nail‐varnish imprint.

#### Wax removal

2 Fill a 250‐ml beaker with distilled water and add 3 drops of mild washing‐up liquid.
3 Heat the filled beaker to 80°C in a temperature‐controlled water bath and keep the temperature stable.
4 Take your sample (single conifer needle) with a pair of tweezers, dip it into the beaker, and move it around gently for 1–5 sec (see Table 2 for species‐specific advice; see Figure 4A–C).
  Take special care to avoid visible tissue damage, such as heat‐induced discoloration (yellowing) or loss of structural integrity.

**Table 2:** Species‐specific water‐bath exposure times prior to imprint preparation

Species	Exposure time (sec)	Comment
*Cedrus deodara, Larix decidua, Tsuga canadensis*	1	Low wax coverage and heat‐sensitive tissue; brief exposure sufficient
*Picea abies, Picea sitchensis, Pinus nigra, Pinus ponderosa, Pinus sylvestris, Pseudotsuga menziesii, Taxus baccata*	3	Intermediate wax deposition and moderately sunken stomata; moderate exposure required
*Abies alba, Abies grandis*	5	Thick wax layer and deeply sunken stomata; prolonged exposure required for adequate wax removal

**Figure 4:**
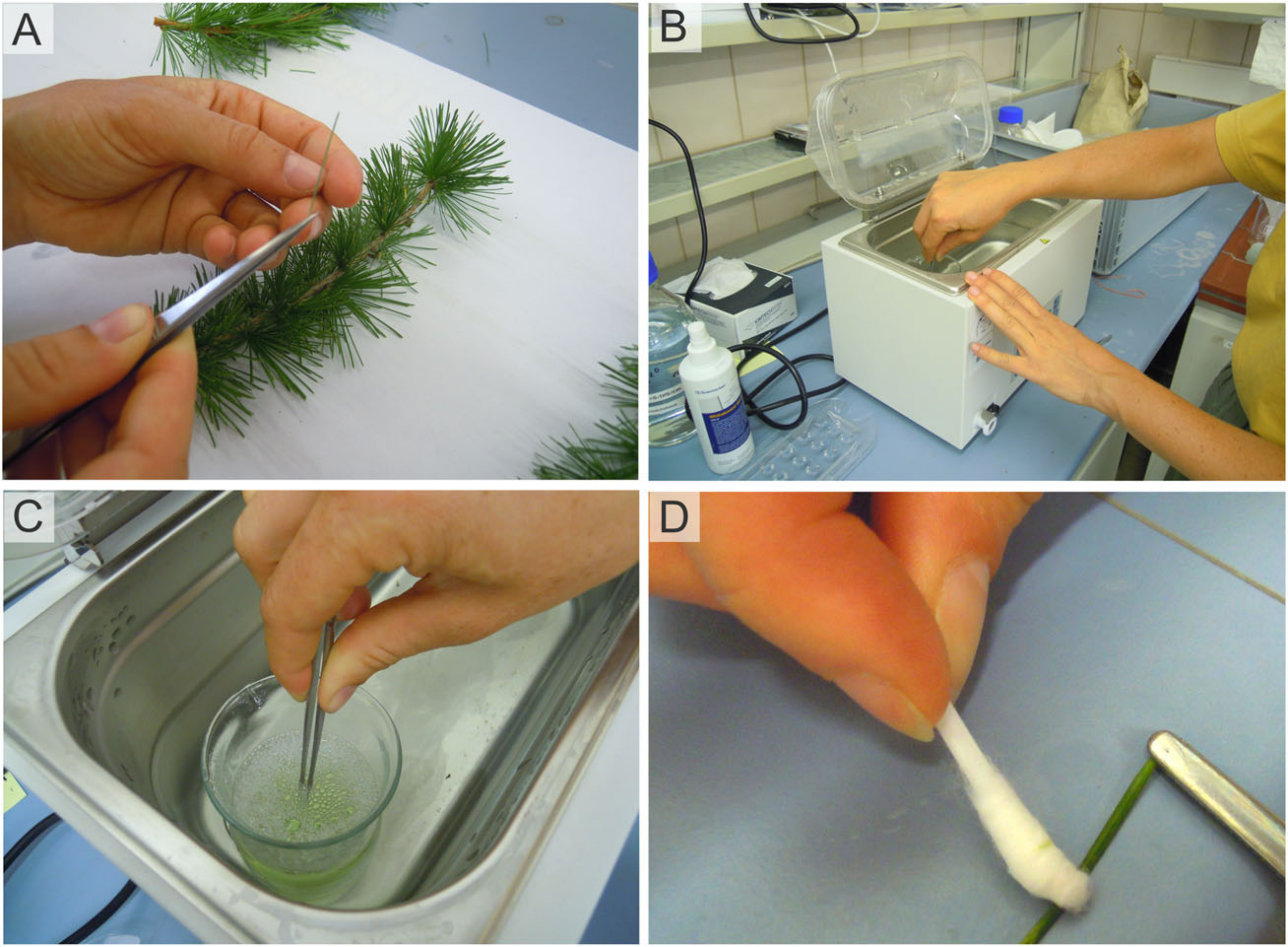
Removal of epicuticular wax from conifer needles prior to imprint preparation. (A) Selection of a single needle sample (here: *Larix decidua*) using tweezers. (B) Immersion of the needle in a beaker of distilled water with mild detergent, held at 80°C in a water bath. (C) Gentle agitation of the needle in the solution to remove surface wax. (D) Blotting the needle (here: *Cedrus deodara*) with a cotton swab to remove residual moisture and detached wax.

5 Gently blot the needle surface dry using a cotton swab to remove residual moisture and remaining wax traces (see Figure 4D).
6 Inspect the needle under a stereomicroscope for remaining white wax residues; if necessary, repeat steps 3–5.

#### Imprint preparation

7 Secure the needle to the work surface using adhesive tape.
8 Apply a thin, even layer of transparent nail varnish onto the needle surface (Figure 5A).

**Figure 5:**
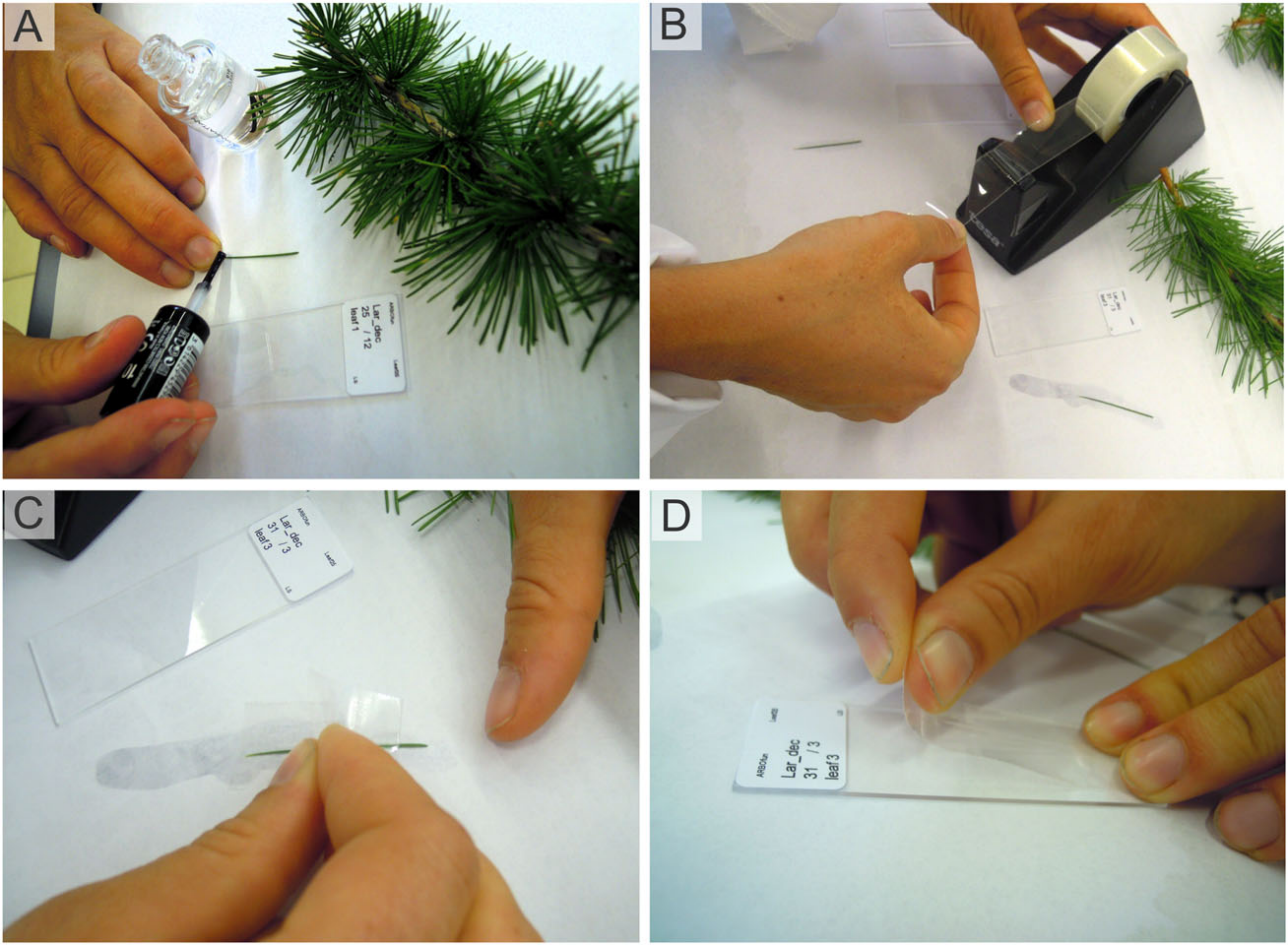
Preparation of epidermal imprints from conifer needles (here: *Larix decidua*) using the nail varnish‐tape method. (A) Application of a thin, even layer of transparent nail varnish onto the immobilized needle surface. (B) Placement of an adhesive tape strip onto the fully dried nail varnish layer. (C) Removal of the tape, lifting the nail varnish imprint from the needle surface. (D) Mounting the tape with the attached imprint onto a labelled microscope slide and smoothing to remove air bubbles.

9 Allow the nail varnish to dry completely.
  Drying takes at least 10 min, depending on the species and ambient laboratory conditions.
10 Place a strip of adhesive tape onto the dried nail varnish layer and press it firmly to ensure full contact with the needle surface (Figure 5B).
11 Carefully peel off the tape, ensuring that the nail varnish layer is fully removed and remains attached to the tape (Figure 5C).
12 Mount the tape strip onto a microscope slide (labelled with the sample ID) and smooth it out gently to eliminate any air bubbles (Figure 5D).
13 Inspect the mounted imprint under a light microscope for completeness of the target epidermal surface and integrity of the replica.
  A high‐quality imprint has clearly visible stomata, without pull‐out artefacts, air bubbles, or other major defects (Figure 9). If quality is insufficient, repeat steps 4–12 with a new sample.
14 Store prepared microscope slides in a dust‐free slide box at room temperature until further analysis.
15 Proceed with stomatal density and size measurements according to Basic Protocol 2.

## Basic Protocol 2 MEASUREMENT OF STOMATAL DENSITY AND SIZE

This protocol outlines the procedures for measuring stomatal density and size from mounted needle imprints using light microscopy and digital image analysis. Users will first acquire low‐magnification (100×) images to systematically count stomata and calculate surface‐specific densities, followed by high‐magnification (1000×) imaging to quantify localized cellular dimensions such as guard cell length (GCL), guard cell pair width (GCPW) and stomatal complex length (SCL), and stomatal complex width (SCW). Properly executed, this protocol ensures consistent, standardized quantification of conifer stomatal traits across varying needle morphologies.

### Materials

Microscope slides with the mounted imprints (Basic Protocol 1)
Stage micrometer
Light microscope equipped with a digital camera (e.g., Leica ICC50 W, using LAS EZ software for imaging)
Image analysis software (e.g., ImageJ)

#### Image Acquisition for Stomatal density

1 Place the microscope slide containing the mounted imprint under a light microscope equipped with a calibrated digital camera.
2 Set the optical magnification to 100×.
3 Record the exact calibrated dimensions of the image captured by the camera software to define the field of view area (FOV_area_).
4 Select three random locations along the length of the needle imprint.
5 Capture images according to the species‐specific arrangement of stomata (see Table 1).
  For species with evenly distributed rows or random stomatal distribution, capture a single, randomly selected image at each of the three locations.
  For all other species: Capture a continuous cross‐section across the entire width of the needle at each of the three locations. If the needle width exceeds a single FOV_area_, capture sequential, non‐overlapping images across the width. If the final image extends beyond the needle margin, the unoccupied background area must be quantified and subtracted during later image analysis.
  For amphistomatic species: Repeat this entire sampling strategy separately for both the abaxial and adaxial surfaces (e.g., Picea sitchensis).

#### Image Analysis for Stomatal Density

6 Open the captured images in image processing software (e.g., ImageJ).
7 Count the stomata per picture.
  Stomata intersecting the image boundary should be included when more than half of the stoma lies within the field of view.
  Overlaying a marker on each counted stoma using a digital marking tool ensures accuracy and prevents double counting.
8 For narrow needles where the imprint does not cover the entire image frame, measure the uncovered background area (A_uncovered_) and subtract it from the total image area (A_total_) to determine the actual sampling area (A_sample_).
$$A_{sample}=\;A_{total\;}-\;A_{uncovered}$$
  If the imprint covers the entire image frame, A_sample_ is just the FOV_area_.
9 Calculate the stomatal density for the stomata‐bearing surface (SD_sbs_) by dividing the total stomata count by the true sample area.
$$SD_{sbs}=\frac{Count}{A_{sample}}$$
10 Calculate the stomatal density of the full surface (SD_fs_; including stomata‐free surfaces) by factoring in both surfaces of the needle:
$$SD_{fs}\;=\frac{Count_{abaxial}+\;Count_{adaxial}}{A_{abaxial}\;+\;A_{adaxial}}$$
  For needles where only one stomata‐bearing surface was sampled, estimate the full needle surface area as twice the sampled area (2 × A_sample_), assuming that the corresponding opposite surface has the same area. Calculate SD_fs_ by dividing the total stomatal count by this estimated full surface area. For species for which both adaxial and abaxial surfaces were sampled, use the combined sampled area of both surfaces. For hypostomatic species, stomatal counts from the adaxial surface are set to 0. For species with stomata evenly distributed over the whole needle surface, SD_fs_ is equal to SD_sbs_.

#### Image Acquisition for Stomatal Dimensions

11 Set the optical magnification to 1000×.
12 Capture one image per stoma at each of the three randomly selected locations along the length of the needle imprint, choosing the stoma that is most centered within the field of view.
  Precise focus on the guard cells is critical. Because stomata are three‐dimensional structures and their appearance changes with the focus level, having a single observer carry out all image acquisition maintains a consistent focal plane.
13 Calibrate the microscope software to permanently embed the scale bar into the image file before saving.
  Alternatively, record the FOV_diameter_ of the 1000× magnification image to use it later as a scale.

#### Image Analysis for Stomatal Dimensions

14 Open the image in image processing software (e.g., ImageJ).
15 Set the global scale in the software according to the length of the embedded scale bar to ensure all subsequent measurements are accurately quantified in micrometers (µm).
16 Use the software's measurement tool to quantify GCL and GCPW.
  See Figure 6 for visual support for the measurements.

**Figure 6:**
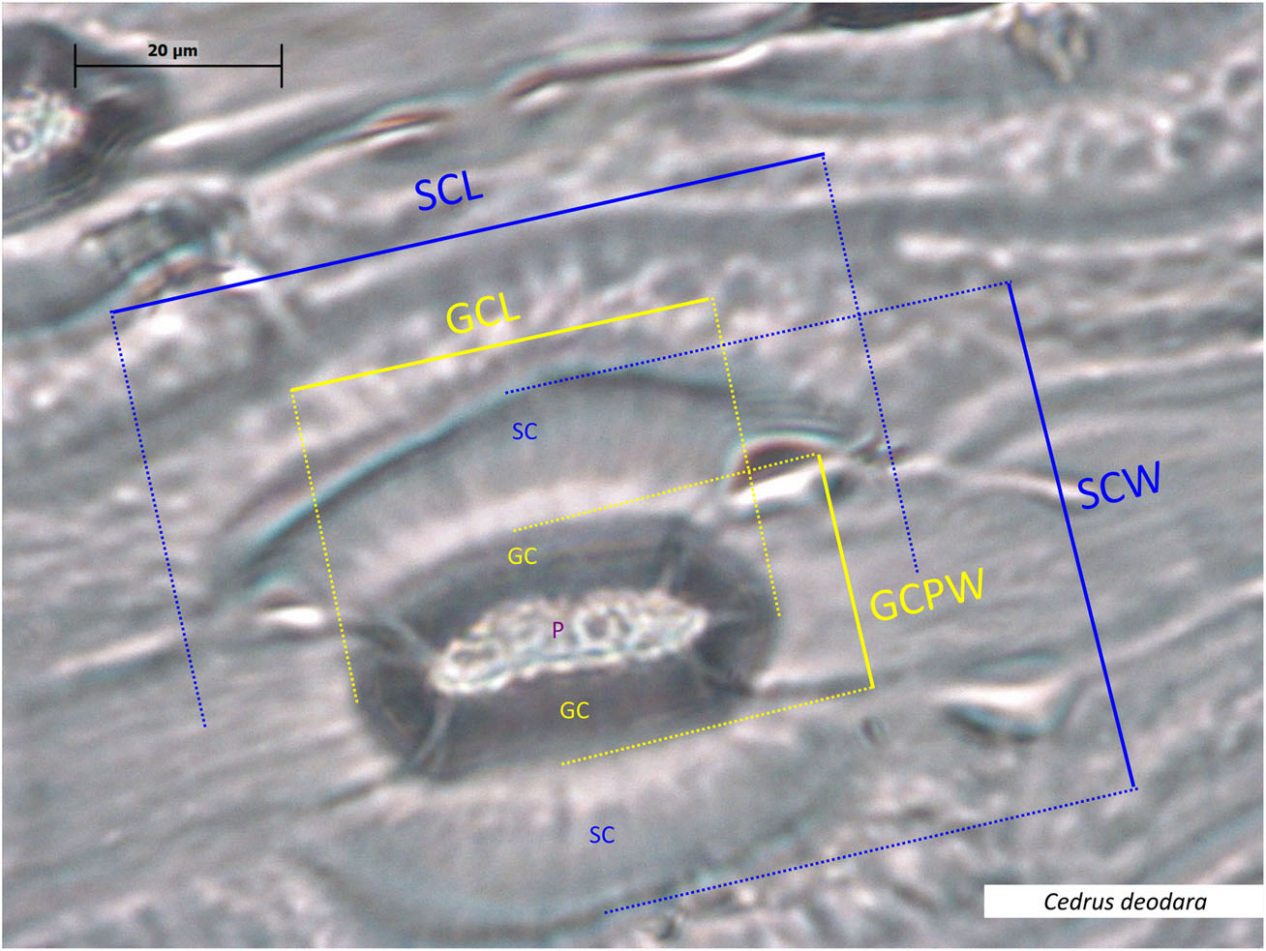
Measurement of guard cell length (GCL) and guard cell pair width (GCPW), as well as stomatal complex length (SCL) and stomatal complex width (SCW), illustrated using a *Cedrus deodara* nail varnish imprint. Image was captured at 1000× magnification using a light microscope equipped with a digital camera (Leica ICC50 W) and LAS EZ imaging software. GC = guard cell, SC = subsidiary cell, P = pore aperture.

17 Measure the SCL and SCW.
  Guard cells frequently overlap or are completely obscured by the surrounding subsidiary cells (sometimes forming a distinct Florin ring; Figure 7) in many conifer species; therefore, GCL and GCPW values can be systematically underestimated.

**Figure 7:**
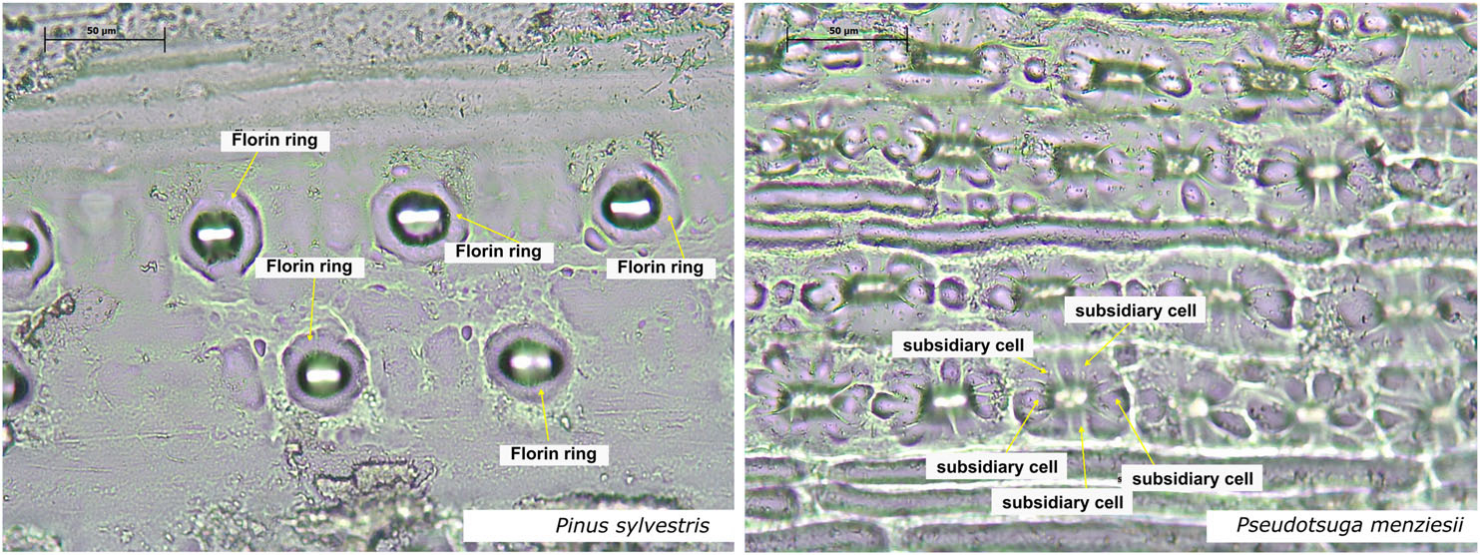
Subsidiary cells of *Pinus sylvestris*, forming Florin rings (left), and of *Pseudotsuga menziesii* (right). Images were captured at 400× magnification using a light microscope equipped with a digital camera (Leica ICC50 W) and LAS EZ imaging software.

18 Save the image together with all measurement data and metadata (e.g., sample ID, magnification, scale) in a clearly structured archive for later quality control and potential re‐evaluation of outliers.

## Commentary

### Critical Parameters

The quality of the epidermal imprint depends on the condition of the sampled leaf tissue. Senescent, damaged, dehydrated, or mechanically distorted tissues can produce incomplete or distorted imprints, making reliable measurements difficult or even impossible. The short hot‐water treatment is intended to remove epicuticular waxes and does not visibly affect epidermal cell or stomatal morphology when applied for the recommended duration. Overexposure, however, can soften or degrade the tissue and may result in poor‐quality imprints.

The most critical step in the protocol is typically the imprinting procedure itself. Although the method appears straightforward in principle, it requires careful and experienced handling, particularly for species with narrow or structurally delicate leaves (e.g., *Larix, Cedrus*). It is essential to capture the full leaf width to avoid bias in stomatal density estimates. This requires a uniform, sufficiently thin application of nail varnish up to and including the leaf margins, adequate drying time, and thorough, even application of adhesive tape (i.e., ensuring full surface contact and proper wrapping) to reliably transfer the entire epidermal surface. Due to these sensitivities, a training and testing phase is strongly recommended for each species prior to large‐scale data collection to ensure that the imprinting procedure yields complete and representative epidermal replicas.

Measurements are sensitive to image acquisition settings and observer decisions during image capture. To minimize variation, all images should be acquired by a single trained observer using consistent microscope settings, with particular attention to magnification, depth of field, and image sharpness, as slight differences in focus can affect the perceived boundaries of stomatal cell structures (Finsinger & Tinner, 2020). Standardization at the imaging stage is therefore critical, as it determines the level of structural detail recorded. Care should also be taken to avoid selection bias during image acquisition, as the search for well‐resolved stomata may unintentionally favour larger and more easily visible stomata, potentially biasing trait estimates.

The treatment of stomata intersecting image boundaries is another methodological decision that should be explicitly reported. In the present protocol, stomata intersecting the image boundary are counted when more than half of the stoma lies within the field of view. A well‐established, systematic stereological approach is to use an unbiased counting frame in which two boundaries are defined as inclusion lines and two as exclusion lines, such that stomata touching the inclusion lines are counted, whereas those touching the exclusion lines are not (Gundersen, 1977). Regardless of the approach chosen, the boundary‐counting criterion should be stated explicitly to allow reproducibility and comparability among studies.

Once the image acquisition and counting criteria are clearly defined, subsequent measurements are less sensitive to observer identity and can be reliably performed by trained assistants, provided the same measurement criteria are applied consistently.

In species possessing subsidiary cells that partially obscure guard cell boundaries (Figure 7), guard cell dimensions are likely to be underestimated. However, because guard cell dimensions are generally correlated with stomatal complex dimensions, the resulting bias is often systematic rather than random (Figure 8). Reporting both metrics is therefore recommended, as stomatal complex measurements can serve as a complementary proxy when guard cell dimensions cannot be reliably resolved.

**Figure 8:**
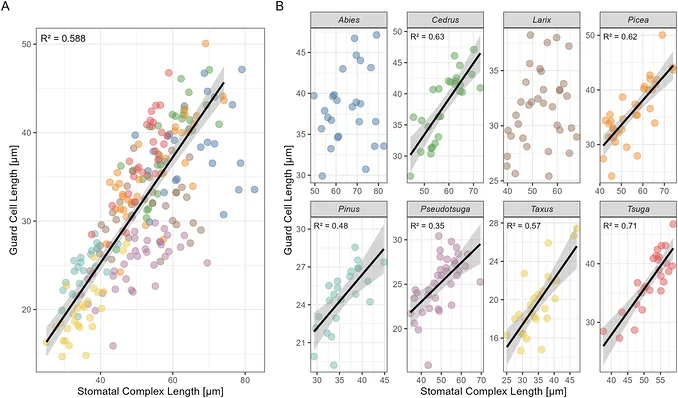
Correlation between Guard Cell Length and Stomatal Complex Length, (A) across genera and (B) within genera. Solid regression lines indicate statistically significant relationships, while non‐significant relationships are shown without regression lines. Coefficients of determination (R²) are provided for all significant models.

The stomatal traits measured in this protocol are not directly comparable to the highly detailed anatomical measurements used in taxonomic or palaeobotanical studies, which resolve fine‐scale structural features at a much higher level of precision (Finsinger & Tinner, 2020; Hu et al., 2016; Lacourse et al., 2016; Sweeney, 2004). Instead, the present protocol prioritizes functionally relevant stomatal traits, such as stomatal density and overall dimensions, that can be measured easily and reproducibly across a broad range of species. By doing so, the method facilitates the generation of large comparative datasets and cross‐clade analyses for robust ecological applications.

This protocol presents procedures and example results for 12 conifer species. However, similar imprinting and measurement challenges were observed among closely related species within the same genera, suggesting that users may also orient their expectations at the genus level.

### Troubleshooting

The most common issues encountered during epidermal imprinting include incomplete wax removal, insufficient nail varnish infiltration or drying, mechanical distortion of the imprint, and poor image quality caused by excessively thick nail varnish layers (Table 3).

**Table 3:** Troubleshooting guide for epidermal imprints and measurement of stomatal density and size

Problem	Possible Cause	Solution
The leaf tissue is too soft to make an imprint, or it has changed colour	Exposure time in hot water was too long, leading to tissue degradation	Reduce the dip‐in time
		Immediately transfer the needle to cold water after the hot bath to prevent tissue deterioration. Ensure that the wax is fully removed beforehand; otherwise, remaining wax may solidify and form irregularly shaped layers in the imprint.
The imprint does not cover the full needle width, and epidermal cells are ripped off at the margins	Nail varnish was not applied all the way to the margins, or the adhesive tape failed to properly bond with the varnish at the margins	Repeat the imprint. Carefully align the tape to ensure full coverage of the varnished area, press firmly along the margins to secure a strong bond, and peel steadily at a shallow angle to avoid tearing the epidermis.
No guard cells are visible, but only round stomatal complexes without internal structures (Figure 9A)	Wax plugs were not removed before imprinting, obscuring underlying structures	Repeat the washing procedure and verify removal of all wax plugs under a stereomicroscope
The imprint shows holes where the stomata should be (Figure 9B)	Wax plugs were not fully removed before imprinting	Repeat the needle washing and check after each dip‐in under the stereomicroscope, that all white wax plugs were removed
	Excessively sunken stomata require better infiltration of nail varnish	Apply a very thin layer of nail varnish, let it dry, then apply a second very thin layer of nail varnish and let it dry
	Insufficient nail varnish drying time before mounting, especially within stomatal pores	Allow additional drying time until the varnish is fully hardened
Imprint appears distorted; epidermal cells are curved or misaligned (Figure 9C), potentially biasing stomatal measurements	The tape was not mounted flat on the microscope slide, causing mechanical distortion	Repeat the imprint. Mount the adhesive tape slowly and as flat as possible onto the microscope slide. Gently smooth the tape during mounting to prevent wrinkles and ensure even adhesion.
Large bubbles with thick, defined margins obscure stomata (Figure 9D)	Air pockets formed between layers (tape–slide or varnish–tape interface)	Smooth the tape carefully during mounting to remove trapped air. Apply only very thin layers of nail varnish. Do not shake the nail varnish bottle before use, as this introduces air bubbles.
The imprint appears blurry or out of focus under the microscope	The nail varnish layer applied to the tissue was too thick, resulting in a deep, distorted impression that prevents clear light transmission	Repeat the application, ensuring a very thin, even layer of varnish is applied. Tip: Store nail varnish in the refrigerator when not in use; keeping it cool lowers its viscosity, allowing it to spread more thinly and evenly.

**Figure 9:**
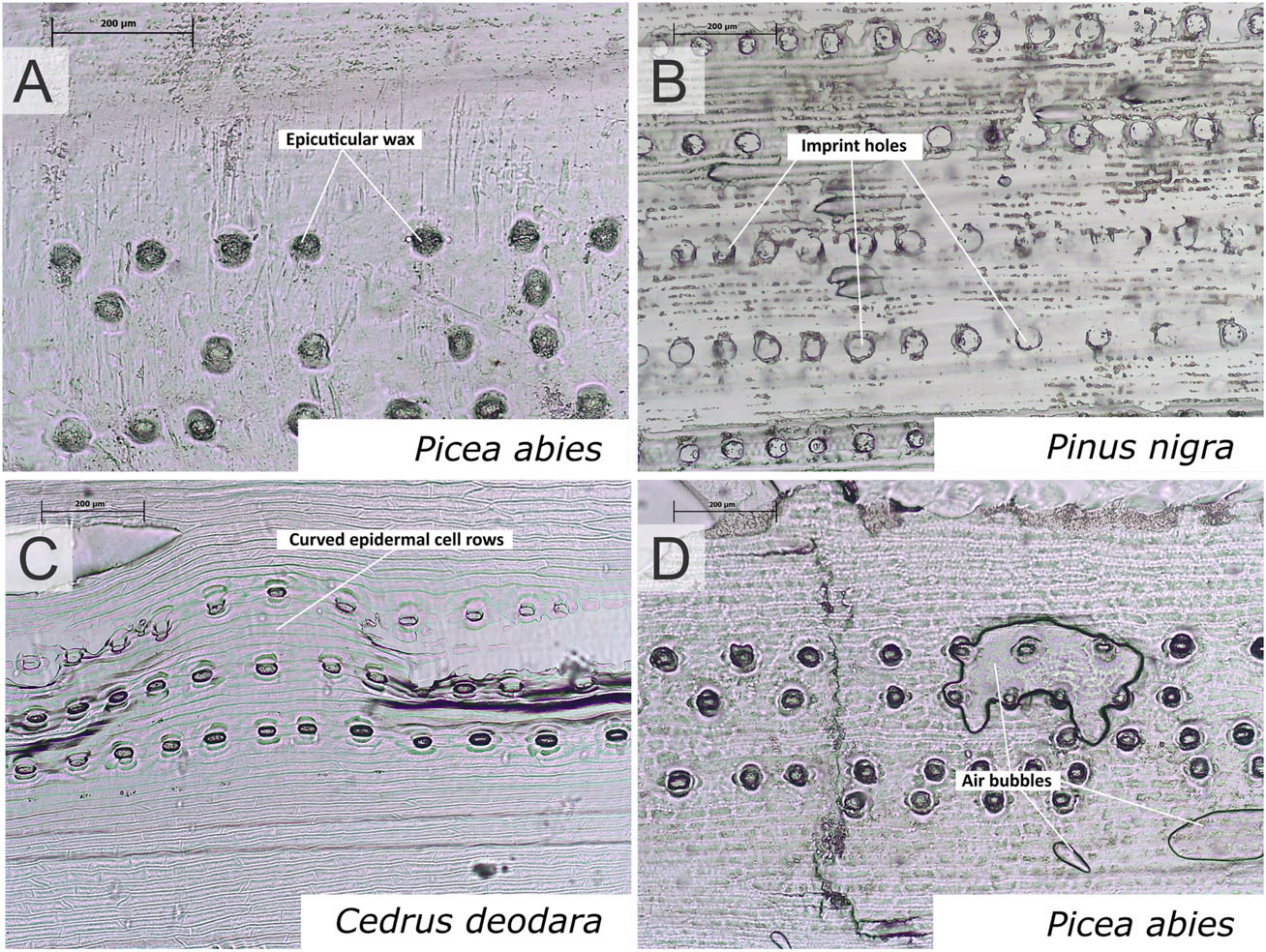
Troubleshooting: Imprints that need to be repeated, due to (A) insufficient removal of epicuticular wax plugs that obscure elements of the stomatal complex, (B) holes in the imprint at the locations where stomata have been, (C) curved epidermal cell rows that indicate that the imprint was not mounted flat enough on the microscope slide, and (D) air bubbles obscuring or biasing underlying cell structures. Images were captured at 100× magnification using a light microscope equipped with a digital camera (Leica ICC50 W) and LAS EZ imaging software.

### Understanding Results

This protocol generates quantitative stomatal traits based on epidermal imprints of conifer needles. To provide a reference for correct implementation and data interpretation, we present a representative dataset of 12 conifer species. Leaf material was sampled in early summer 2025 in the research arboretum ARBOfun in Großpösna, Germany (51°16′ N, 12°30′ E). For each species, samples were collected from three trees, all planted between 2012 and 2014. Three needles were sampled from each tree, resulting in a total of nine imprints per species. From each imprint, three stomata were measured for dimensional analysis, and three positions were used to count stomatal density.

Figure 10 illustrates typical outputs from the imprinting and imaging procedure at 100× magnification, highlighting stomatal distribution patterns across the needle surface. Figure 11 shows images at 1000× magnification, showing individual stomatal complexes used for dimensional trait measurements. Together, these images serve as a visual reference for acceptable imprint quality.

**Figure 10:**
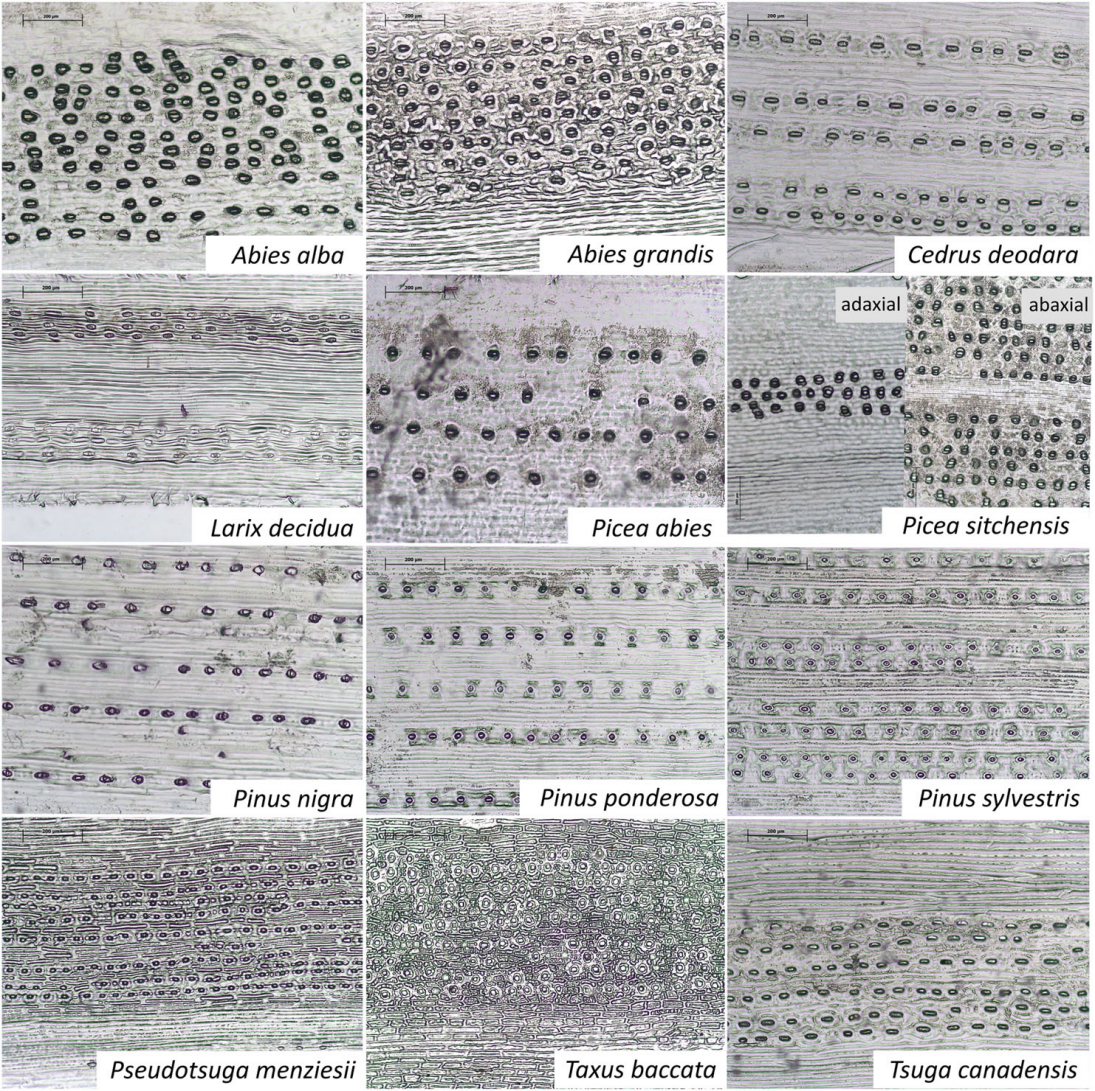
Stomatal arrangement patterns of 12 conifer species. Images were captured at 100× magnification using a light microscope equipped with a digital camera (Leica ICC50 W) and LAS EZ imaging software. Only stomata‐bearing surfaces are shown. For all species except *Picea sitchensis*, this represents either the abaxial surface or, for species with stomata distributed around the needle circumference, a randomly selected portion of the needle surface. For *Picea sitchensis*, both the adaxial and abaxial stomata‐bearing surfaces are shown.

**Figure 11:**
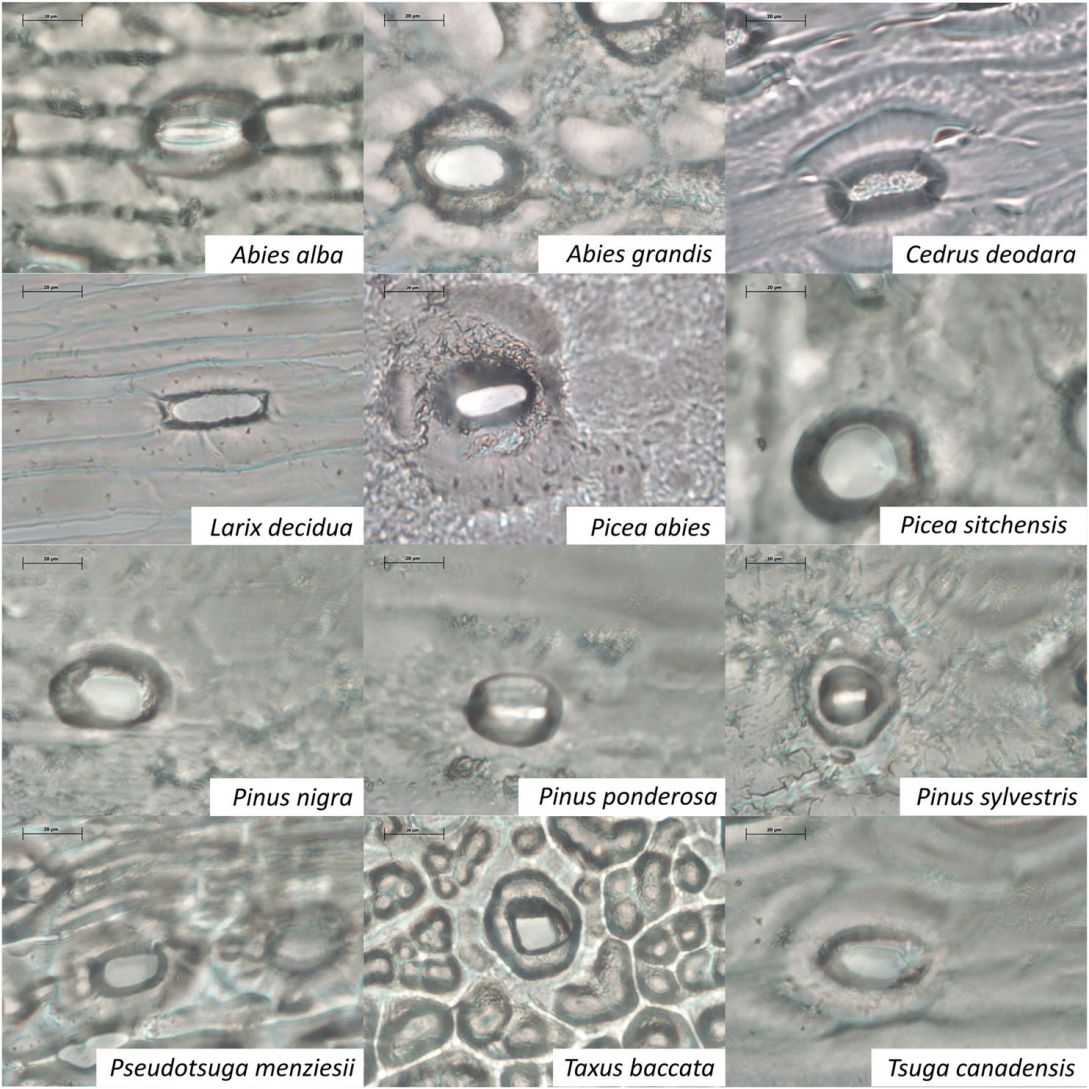
Close‐up images of stomata from 12 conifer species. Images were captured at 1000× magnification using a light microscope equipped with a digital camera (Leica ICC50 W) and LAS EZ imaging software.

Based on these imprints, we quantified stomatal density (SD) across the entire (full) needle surface (SD_fs_) and on the stomata‐bearing surface only (SD_sbs_; Figures 3 and 12). Density values calculated across the full needle surface are consistently lower for species with stomata‐free surfaces or highly asymmetric densities between surfaces (e.g., *Picea sitchensis*). In contrast, species with evenly distributed stomata (e.g., *Cedrus deodara, Larix decidua, Picea abies, Pinus spp.)* show no difference between SD_fs_ or SD_sbs_. The definition of the surface area considered should therefore be explicitly stated when comparing studies or species.

**Figure 12:**
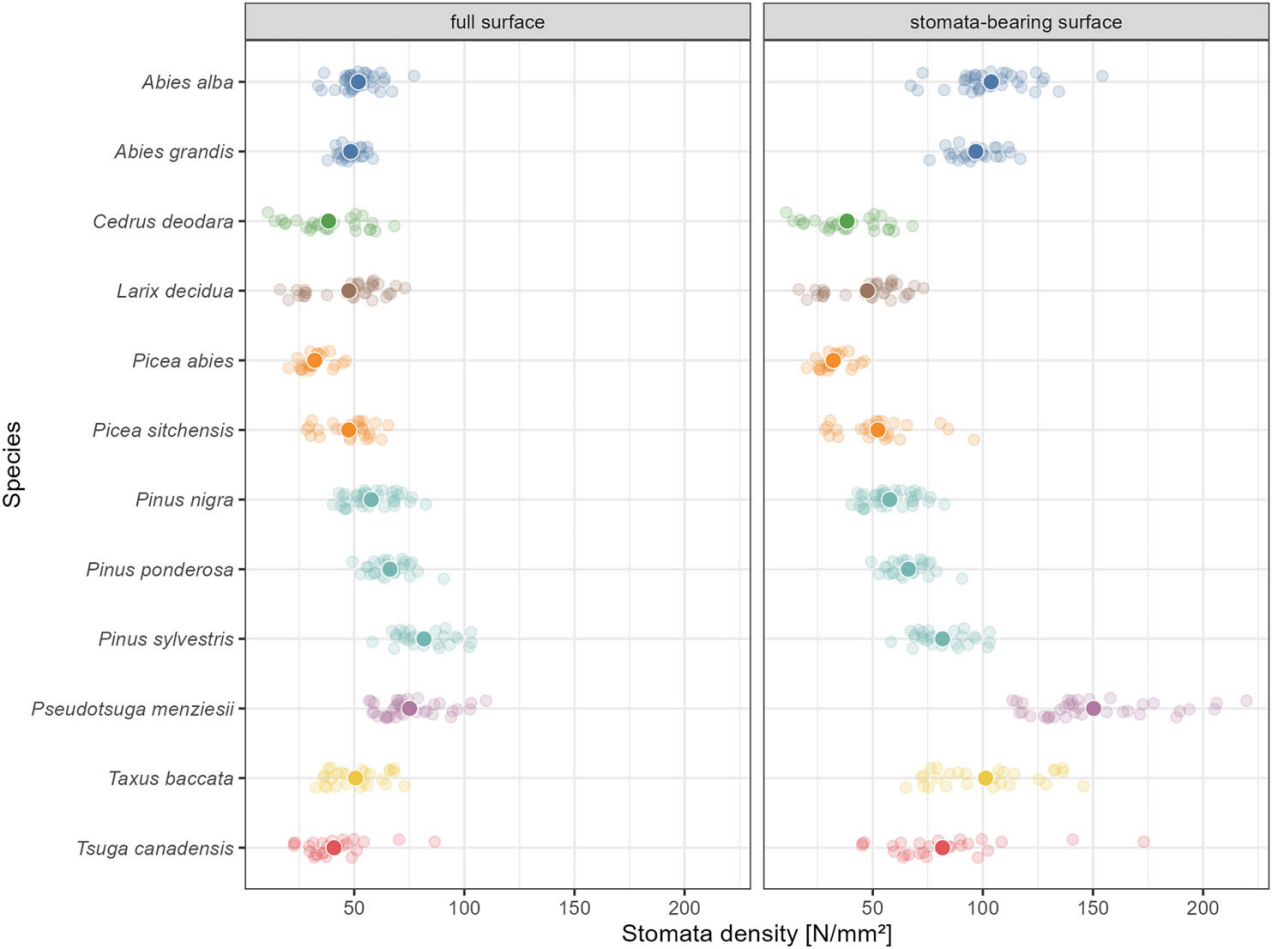
Stomatal density of 12 conifer species, calculated per either full surface (left panel) or only on stomata‐bearing surface (right panel). Values are identical for species with stomata distributed across the entire leaf surface. Solid points indicate species means. Individual points represent measurements from a hierarchical sampling design comprising three trees per species at the ARBOfun Research Arboretum (Großpösna, Germany; 51°16′ N, 12°30′ E), three needles from different upper‐canopy branchlets per tree, and three measurement positions per needle.

Stomatal size traits, including guard cell length (GCL), guard cell pair width (GCPW), guard cell area (GCA), stomatal complex length (SCL), stomatal complex width (SCW), and stomatal complex area (SCA), show clear species‐specific differences (Figure 13). The hierarchical sampling design further demonstrates that variation among needles and tree individuals within a species is generally smaller than variation among species for both stomatal density and guard cell length (Figure S1), indicating that the protocol yields consistent measurements across biological replicates. For some species, we found tree individuals to be quite distinct in their stomatal density patterns (e.g., *Larix decidua, Taxus baccata*), reflecting either intraspecific phenotypic plasticity driven by microenvironmental differences or underlying genotypic variation among individuals.

**Figure 13:**
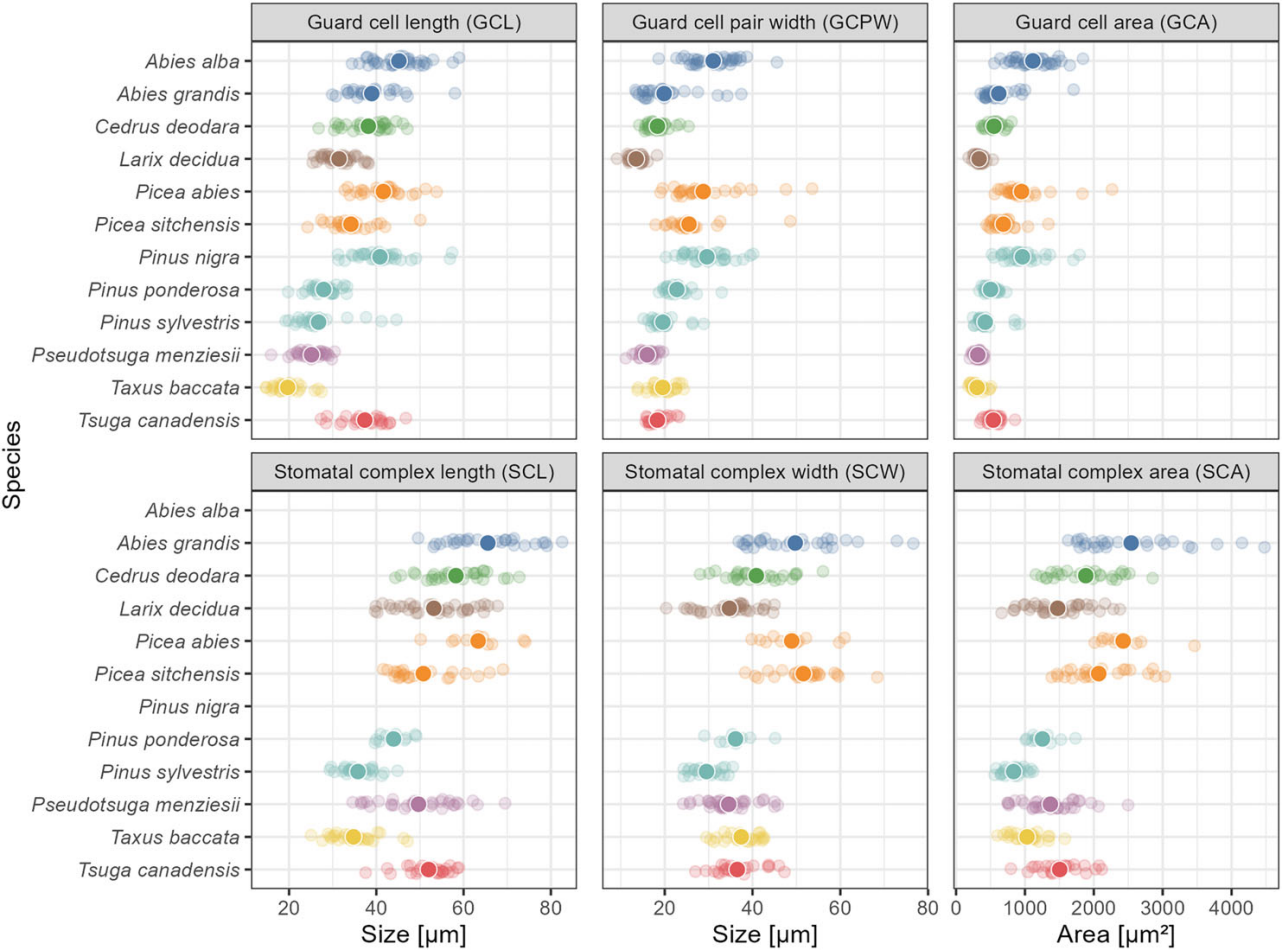
Data of stomatal size measurements. Upper panels: Guard cell dimensions (length, width, and area). Guard cell area (GCA) was calculated assuming an elliptical shape ($\mathrm{GCA}=\;\pi \times \frac{\mathrm{GCL}}{2}\times \;\frac{\mathrm{GCPW}}{2}$). Lower panels: Dimensions of the whole stomatal complex (length, width, and area, calculated using the same elliptical formula). Stomatal complex data are omitted for *Abies alba* and *Pinus nigra* because subsidiary cell borders could not be distinguished with sufficient certainty from epidermal cells on the imprints. Solid points indicate species means. Individual points represent measurements from a hierarchical sampling design comprising three trees per species at the ARBOfun Research Arboretum (Großpösna, Germany; 51°16′ N, 12°30′ E), three needles from different upper‐canopy branchlets per tree, and three measurement positions per needle.

Variation among replicate measurements within a single needle likely reflects a combination of measurement uncertainty and small‐scale biological variability. For stomatal density, differences among replicate images may partly reflect natural within‐needle variation in stomatal distribution, e.g., a tendency for fewer stomatal rows towards the needle tip, which can influence estimates depending on sampling position. For stomatal size traits, we sometimes observed differences within the same needle, indicating genuine within‐needle heterogeneity. At the same time, some of the observed variations may still be attributable to measurement uncertainty and minor differences in the selection and positioning of individual stomata during image acquisition. We recommend measuring more stomata per imprint when fine‐scale variation is of interest.

At the species level, stomatal density (SD_fs_) and guard cell length (GCL) showed a weak negative relationship (Figure 14), with species having higher stomatal densities tending to have smaller guard cells (p = 0.06; R² = 0.31). One notable exception was *Taxus baccata*, which deviated from this general trend by combining very small stomata with intermediate stomatal density. This deviation may be linked to its distinctive foliar anatomy among conifers, including a stomatal distribution that is not linear but resembles angiosperm‐like patterns (Figure 10). Within species, only *Tsuga canadensis* and *Pinus ponderosa* showed a marginal relationship between density and size, whereas all other species showed no detectable association.

**Figure 14:**
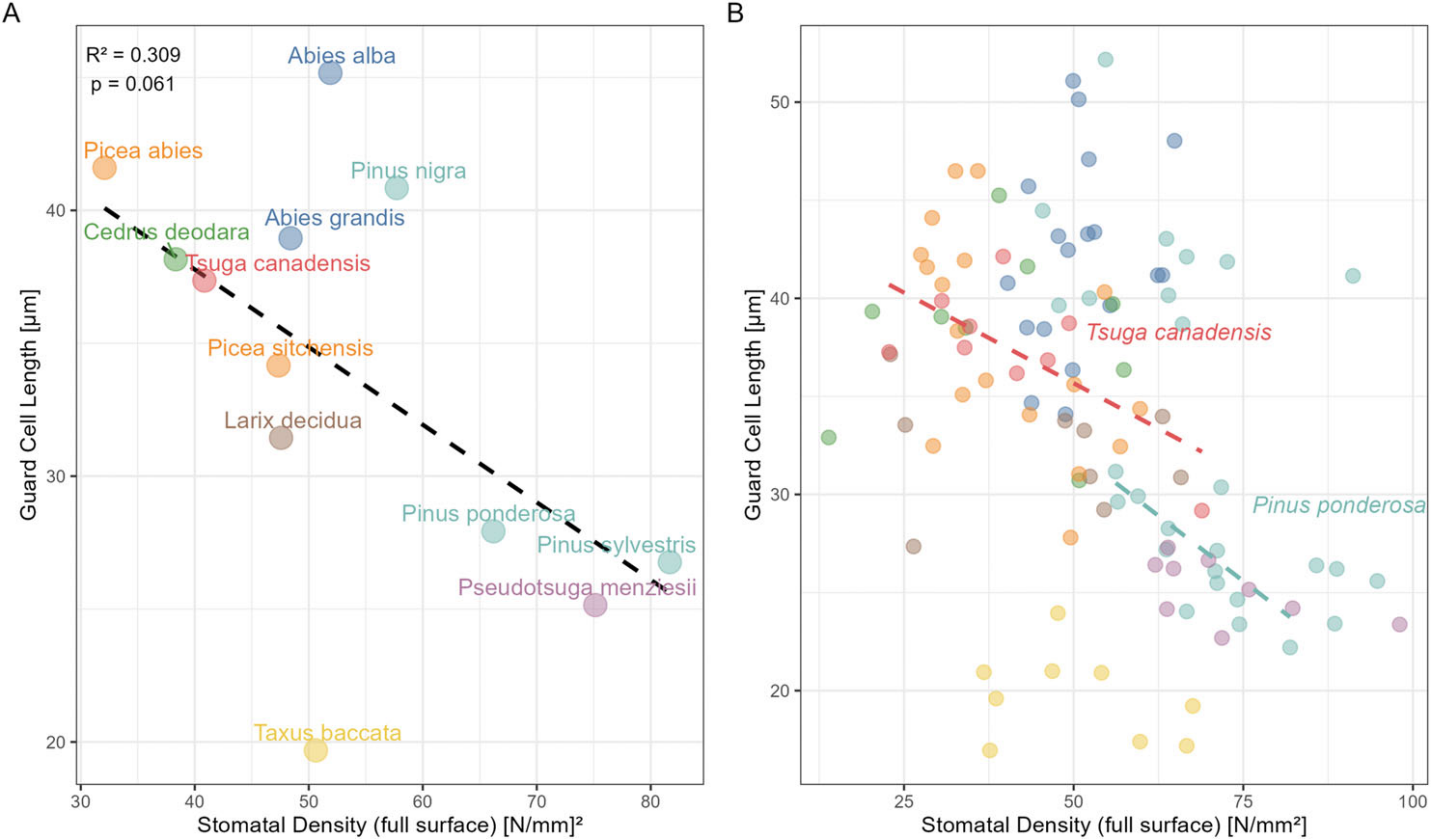
Relationship between stomatal density (SD_fs_) and guard cell length (GCL) across species (A) and within species (B). Dashed lines indicate linear regression fits with marginal statistical significance.

### Time Considerations

The time required to produce a single epidermal imprint varies strongly by species. For species that are straightforward to process (e.g., *Abies alba, Abies grandis, Taxus baccata*, and *Tsuga canadensis*), trained personnel can typically obtain a high‐quality imprint in approximately 3 min of working time (excluding ∼10 min of nail varnish drying time). For more challenging species, the procedure may require up to 15 min of working time. Increased processing times may result from deeply sunken stomata that require longer drying times (e.g., *Pinus spp*.), the need to sample both adaxial and abaxial needle surfaces (e.g., *Picea sitchensis*), or the careful handling required for particularly thin and delicate needles (e.g., *Larix decidua* and *Cedrus deodara*). Additional time may be required when imprints need to be repeated due to insufficient quality, when multiple thin layers of nail varnish are necessary to prevent stomatal structures from tearing out of the imprint (*e.g., Pinus spp*.), or when wax removal requires iterative hot‐water treatments with repeated inspection under a stereomicroscope. In general, species with narrow needles, deeply sunken stomata, or persistent epicuticular wax deposits require the most processing time and effort. A preliminary test phase for each target species is strongly recommended before initiating large‐scale sampling. This step calibrates processing times to species‐specific material properties and laboratory‐ and operator‐specific handling conditions, resulting in more realistic time estimates for the main campaign.

When imprint quality is high, acquiring three images per imprint (at 100× magnification) for later stomatal density analysis is generally rapid. However, processing time increases for species with needle widths that exceed the microscope's field of view, as two to three images per sampling position are required to capture the full needle width. Acquiring three images per imprint (at 1000× magnification) for stomatal dimension analysis may be more time‐consuming, as repeated scanning and repositioning are often required to identify well‐focused stomata with clearly visible structural details. Overall, we estimate a total microscopy time of approximately 7–10 min per slide, including image acquisition from three positions at 100× magnification and three individual stomata at 1000× magnification.

Counting stomata in individual images is a relatively fast and straightforward task that can be performed reliably by minimally trained personnel. This step is also readily amenable to partial or full automation, which may further reduce processing time. For manual counting, approximately 1 min per image is required. Given 3 (up to 6–9 when two to three images are required to cover the needle width due to field‐of‐view limitations) images per imprint, this corresponds to approximately 3–9 min per imprint. In contrast, quantitatively measuring stomatal dimensions is more time‐intensive, requiring approximately 3 min per image and resulting in an estimated 9 min per imprint. Prolonged visual inspection may lead to operator fatigue and reduced measurement accuracy; therefore, regular breaks are recommended to maintain consistent data quality.

### Author Contributions

**Lena Sachsenmaier**: Conceptualization; data curation; formal analysis; investigation; methodology; project administration; visualization; writing—original draft. **Selina Franz**: Investigation; methodology; writing—review and editing. **Anja Kahl**: Methodology; writing—review and editing. **Lena Kretz**: Investigation; validation; writing—review and editing. **Christian Wirth**: Funding acquisition; resources; supervision; writing—review and editing.

### Conflict of Interest

The authors declare no competing interests.

## Supporting information


*Figure S1: Variation in (A) stomatal density (stomata‐bearing surface, sbs) and (B) guard cell length (GCL) across the hierarchical sampling design. Boxplots represent the three sampled tree individuals per species, while point shapes indicate measurements from different needles within each tree*.

## Data Availability

The data that support the protocol are openly available in Zenodo at https://doi.org/10.5281/zenodo.21098915, reference number 21098915.
